# Editorial: Legumes for Global Food Security

**DOI:** 10.3389/fpls.2020.00926

**Published:** 2020-07-07

**Authors:** Jose C. Jimenez-Lopez, Karam B. Singh, Alfonso Clemente, Matthew N. Nelson, Sergio Ochatt, Penelope M. C. Smith

**Affiliations:** ^1^Department of Biochemistry, Cell & Molecular Biology of Plants, Estación Experimental del Zaidín, Spanish National Research Council (CSIC), Granada, Spain; ^2^Agriculture and Food, Commonwealth Scientific and Industrial Research Organization (CSIRO), Perth, WA, Australia; ^3^Department of Physiology and Biochemistry of Animal Nutrition, Estación Experimental del Zaidín, Spanish National Research Council (CSIC), Granada, Spain; ^4^Agroécologie, AgroSup Dijon, INRAE, Université de Bourgogne, Dijon, France; ^5^Legumes for Sustainable Agriculture, School of Life Sciences, La Trobe University, Melbourne, VIC, Australia

**Keywords:** legumes, food security, legume breeding, sustainable agriculture, climate resilience, genetic resources, environmental stresses and physiology

Global climatic change combined with population growth is imposing a huge pressure on demand for agronomic resources. These effects are major threats for food security having a great impact on the agroecosystem and generating various abiotic and biotic stresses that, in turn, trigger many physiological and metabolic disorders in plants. These stresses reduce crop yields at precisely the time when they need to increase to reach the demands of the increasing population. One of the major scientific and agronomic challenges of this century is to understand and, when possible, withstand stress so that yields are maintained, even under stressful conditions. This special issue brings together a range of scholarly review and research articles focused on legume crops, key components of healthy diets and productive crop rotations. Here we summarize some of the highlights derived from the 36 articles published in this special issue.

Ribalta et al. provided novel information on the impact of growing conditions on the progress of seed development and maturation, and also analyzed the endogenous hormone accumulation across diverse pea genotypes, thereby providing further insights into the mechanism of hormonal regulation of legume seed development and *in vitro* precocious germination.

Rani et al. have reviewed the literature relevant to the development of climate-resilient chickpea through the exploitation of biotechnological and molecular approaches for the generation of novel genotypes with an improved resistance to extreme temperatures and drought.

Basu et al. took a physiological approach to explore heat tolerance and grain filling in *Vigna radiata*. They measured response to heat stress during the sensitive reproductive phase over 3 years in two field locations in a panel of 116 accessions. They focused on a subset of 17 contrasting accessions to perform heat stress experiments in controlled glasshouse conditions. The most promising accessions could be distinguished using a set of 11 PCR-based markers. Further work will be required to explore the genetics of heat tolerance during reproduction in this species.

Nair et al. have comprehensively reviewed the abiotic and biotic stresses that affect *Vigna radiata*, many that will be relevant in a climate change situation, and addressed the challenges for breeding of more resilient lines. Further breeding utilizing the available molecular technologies will be essential to make the most of the advantages of this legume that is an important source of protein for human nutrition.

Working with peanut, Tian et al., examined the effect on salinity tolerance of priming 4-week-old seedlings with the green volatile (Z)-3-Hexeny-1-yl acetate (Z-3-HAC), comparing one salt-sensitive and one salt-tolerant peanut genotype. Z-3-HAC primed seedlings exhibited increased relative water content, net photosynthetic rate, maximal photochemical efficiency of photosystem II and activities of the antioxidant enzymes; moreover, osmolyte accumulation under salt stress and coupled with significantly reduced reactive oxygen species, electrolyte leakage, and malondialdehyde content compared to non-primed plants. Z-3-HAC also increased the total length, surface area, and volume of roots under salt conditions. Thus, Z-3-HAC generated a priming-induced modification of the photosynthetic apparatus, antioxidant systems, osmoregulation, and root morphology protecting the peanut seedlings from salinity.

Working with *M. truncatula* and tobacco cell suspensions, Elmaghrabi et al. observed that under high NaCl levels both cell and nuclear size decreased but were not useful markers of cell survival under salt stress, while nuclear marginalization, observed for the first time concomitant with salinity in plant cells, could be a novel and helpful morphological indicator for acquisition of salinity tolerance and may be a common response across eukaryotes.

Legume crops are valued in crop rotations in part due to their ability to raise soil N levels through symbiotic nitrogen fixation (SNF) with rhizobial species. Although extreme temperatures may have detrimental effects on growth and development, alfalfa is a legume crop known for its climate-resilience. Liu et al., studied the role of symbiosis with rhizobium on the plant's performance under low temperature stress conditions, by comparing plants with active or inactive nodules or no nodules at all. They found that plant survival was higher in those with active nodules. Irrespective of whether nodules were active or not, nodulated plants accumulated more soluble proteins and sugars, compared to plants without nodules, which exhibited a greater activity of oxidation protective enzymes; rhizobia nodulation enhanced the tolerance of plants to low temperatures through an alteration of the expression of regulatory and metabolism associated genes.

The common use of N fertilizers in modern agriculture has raised the N content of many soils and may have led to weakened selection for SNF efficiency in modern legume breeding. This hypothesis was tested in common bean by Wilker et al., comparing the SNF efficiency and agronomic performance under low soil N levels of 19 modern cultivars (bred under high soil N conditions) to 25 heirloom varieties (bred under lower soil N conditions). There was wide genetic variation for SNF efficiency but on average heirloom bean varieties were not any more SNF efficient than modern cultivars, although the best performer was an heirloom variety. The authors advocated the incorporation of heirloom varieties into modern bean breeding programs.

Phenological adaptation is a key aspect of crop productivity and is highly relevant to food security in the light of changing climates. Furthermore, flowering time is a key trait in breeding and crop evolution, due to its importance for adaptation to different environments and for yield. The molecular control of flowering is now well-understood in the model plant Arabidopsis. However, despite the importance of legumes for food security, there are large gaps in our understanding of how phenology is controlled at the molecular level in legumes. Zhang L. et al. used a transgenic approach to investigate the mode of action of a homologue of the Arabidopsis photoperiod response gene *CDF* in Medicago. Rather than acting to suppress expression of the floral integrator gene *FT* via the photoperiod gene *CO* (as is the case in Arabidopsis), *CDF* appears to directly suppress *FT* independently of *CO* in Medicago.

Ortega et al. analyzed two different inbred populations to examine the genetic control of domestication-related differences in flowering time and growth habit between domesticated chickpea and its wild progenitor *Cicer reticulatum*. A single major quantitative trait locus for flowering time under short-day conditions [*Days To Flower (DTF)3A*] was mapped to a 59-gene interval on chromosome three containing a cluster of three *FT* genes, which collectively showed upregulated expression in domesticated relative to wild parent lines. They point to de-repression of this specific gene cluster as a conserved mechanism for achieving adaptive early phenology in temperate legumes.

The exploitation of hybrid vigor is common across many grain and vegetable crops, yet remains under-exploited in legume crops. Hybrid vigor can increase grain yield, broaden adaptation and improves weed competitiveness. A major hindrance to the development of hybrid legume varieties is the lack of male-sterility systems for hybrid seed production. In this regard, the production of engineered male sterile plants by expression of a ribonuclease gene under the control of an anther, i.e., ENDOTHECIUM 1 (PsEND1),—or pollen-specific promoter has proven to be an efficient way to generate pollen-free elite cultivars. Roque et al. studied the genetic control of flower development in legumes and several genes that are specifically expressed in a determinate floral organ. Using genetic constructs carrying the PsEND1 promoter fused to the uidA reporter gene and to the barnase gene produces full anther ablation at early developmental stages, preventing the production of mature pollen grains in all plant species tested. Additional effects with interesting biotechnological applications include the redirection of resources to increase vegetative growth, the reduction of the need for deadheading to extend the flowering period and the elimination of pollen allergens in ornamental plants. The PsEND1::barnase-barstar construct could also be useful to generate parental lines in hybrid breeding approaches to produce new cultivars in different legume species.

Heat stress during flowering has a detrimental effect on legume seed yield, mainly due to irreversible loss of seed number. In this regard, Liu et al. provided an overview of the developmental and physiological basis of controlling seed setting in response to heat stress, and showing that the entire seed setting process in legume crops including male and female gametophyte development, fertilization and early seed/fruit development is sensitive to heat stress, particularly male reproductive development.

In pea seeds, an important source of protein for food and feed, N partitioning is a key component for seed quality and yield. Lamure and Munier-Jolain investigated the effect of temperature on N partitioning during seed filling. High temperatures have a significant effect reducing the amount of N in mature seeds. This appears to be a result of reduced sink strength for N and reduced duration of seed filling. N seems not to be efficiently remobilized from leaves, being particularly obvious for nitrate fertilized plants, where although more nitrate was assimilated in high temperatures it was not mobilized into the seeds.

Nutrient remobilization was addressed in another study on pea by Gallardo et al. They combined investigation of N remobilization using ^15^N labeling and analysis of transcriptional changes occurring as seed filling progresses to identify possible transcriptional regulators of the process. The authors showed a dynamic remobilization of N from leaves at reproductive and vegetative nodes and later from all organs. Their parallel analysis of the same processes in *M. truncatula* identified regulatory steps that may be shared by both plants.

Cold damage has become the key limiting factor of early sowing. Zhang H. et al. reviewed membrane lipid metabolism and its molecular mechanism, as well as lipid signal transduction in peanut (*Arachis hypogaea* L.) under cold stress to build a foundation for explaining lipid metabolism regulation patterns and physiological and molecular response mechanisms during cold stress and to promote the genetic improvement of peanut cold tolerance.

The multidimensional nature of plant-pathogen interactions and the production of disease-resistant crop plants that are resilient to climate change are major agricultural challenges currently under thorough investigation. The manuscript by Kankanala et al. reviewed how genomic approaches are increasing our understanding of plant-pathogen interactions in legumes. This is an important and timely review given the major losses most legume crops face annually due to disease issues. They comprehensively covered a range of topics in terms of legume crops and diverse pathogens, with a major focus on transcriptomic studies. These studies have greatly expanded in recent years due to the increasing affordability of next generation sequencing approaches and the production of reference genomes for many legume crops, helping to identify a number of potentially key genes for both resistant and susceptible interactions. The review also looked at how genomic approaches will facilitate breeding for resistance to pathogens in legumes by describing some of the molecular tools to incorporate defense related traits into breeding programs.

The manuscript by Nay et al. analyzed disease resistance in common bean to angular leaf spot, an important disease worldwide that is caused by the fungal pathogen, *Pseuocercospora griseola*. They looked at 316 common bean lines representing a diversity set, under both glasshouse and field conditions, with the latter taking place at multiple sites in South America and Africa. They used genotyping by sequencing and genome wide association mapping to study the response of the common bean lines to different races of the pathogen. In contrast to an earlier work, which had identified 5 significant resistant loci, this comprehensive study found only 2 to be important, Phg-2 and Phg-4, with Phg-2 being effective against multiples races.

Das et al. assessed in field pea genotypes the magnitude of environmental and genotype-by-environment interaction on the resistance against rust notably influenced by environmental factors. They identified various “ideal” genotypes as IPF-2014-16, KPMR-936 and IPF-2014-13, which can be recommended for release and exploited in a resistance breeding program for the region confronting field pea rust.

Chaudhari et al. screened a set of 340 diverse peanut genotypes for LLS and rust resistance and yield traits across three locations in India under natural and artificial disease epiphytotic conditions. The study revealed significant variation among the genotypes for LLS and rust resistance in different environments. These data revealed significant environment (E) and genotype × environment (G × E) interactions for both diseases indicating differential response of genotypes in different environments. Pod yield increase as a consequence of resistance to foliar fungal diseases suggests the possibility of considering “foliar fungal disease resistance” as a must-have trait in all the peanut cultivars that will be released for cultivation in rainfed ecologies in Asia and Africa.

Zhou et al. investigated differential expression of 10 Resistance Gene Analogs (RGAs), which are key factors in the recognition of plant pathogens and the signaling of inducible defenses, among cultivated chickpea varieties which are resistant or susceptible to the foliar disease Ascochyta blight caused by the fungus *Ascochyta rabiei* (syn. *Phoma rabiei*). They found significant differential expression of four RGAs that were consistently upregulated in the most resistant genotype, ICC3996, immediately following inoculation, when spore germination began and ahead of penetration into the plant's epidermal tissues. These represent clear targets for future functional validation and potential for selective resistance breeding for introgression into elite cultivars.

Nair et al., reviewed the progress and potential for genetic improvement of mung bean for resistance to biotic stress including fungal and bacterial pathogens, viruses and insects and as for their analysis of abiotic stress discussed the constraints to breeding to overcome these pests and pathogens.

The manuscript by Zwart et al. provided a detailed review of resistance to nematodes in chickpea. A range of nematode pests are major problems for chickpea with combined annual yield losses of around 14% from root-knot, cyst and root-lesion nematodes. Resistance to these nematode species in cultivated chickpea (*Cicer arietinum*) is limited due to the narrow genetic diversity but, as detailed in this comprehensive review, good levels of resistance exist in a number of wild chickpea species. However, barriers to interspecific hybridization hinder the use of some of these wild species as sources of nematode resistance, although others such as *C. reticulatum* and *C. echinospermum* have been valuable sources of nematode resistance genes as well. The review also discusses the use and potential of genome-assisted breeding strategies to improve nematode resistance in chickpea.

Genetic and genomic resources of grain legumes are strategic and valuable tools currently under forefront research worldwide, bringing the knowledge and opportunity to facilitate the identification of specific germplasm, trait mapping and allele mining to more effectively develop biotic and abiotic-stress-resistant and high quality grains for food and feed.

One of the main yield-determining traits under stress conditions is seed weight. In this sense, Karikari et al. have studied the genetic basis of 100-seed weight for the development of new improved soybean cultivars. They evaluated a recombinant inbred line (NJIR4P) in four different environments by using a high density interspecific linkage map which allowed them to detect 19 stable QTLs distributed on 12 chromosomes in all individual environments plus combined environments, seven of which were minor (*R*^2^ <10%) but novel, while eight were stably identified in more than one environment. Of the 12 QTLs detected in this study which co-localized with earlier reported ones having narrow genomic regions, only 2 QTLs were major (*R*^2^ > 10%). Beneficial alleles of all identified QTLs were derived from cultivated soybean parent (Nannong4931). Based on PANTHER (Protein ANalysis through Evolutionary Relationships), gene annotation information, and literature searches, 29 genes within 5 stable QTLs were predicted to be possible candidate genes regulating seed-weight/size in soybean. Although their role in seed development needs further validation, this work underlined the considerable scope still available for the genetic improvement of 100-seed weight in soybean using candidate gene mining and subsequent marker-assisted breeding.

Pea has been studied as genetic model since the Eighteenth century, with key contributions to genetics and the development of the basic principles of heredity. The pea genome is characterized by its large size (~4.45 gigabases) of which ~85% is comprised of highly repetitive sequences. In this topic, Gali, Tar'an et al., reports the construction of a sequence-based physical map of the pea genome using whole-genome profiling (WGP). This study reports a very valuable dataset that will provide a framework to obtain a reference pea genome sequence to further explore the genes governing major traits, including those influencing seed yield and seed quality.

Raggi et al. focused on the genetic control of phenology in common bean. They recorded flowering date in a panel of 192 inbred lines developed from diverse European landraces at two sites over two seasons. They genotyped the panel using a RADseq approach and performed a genome wide association study (GWAS) to identify seven candidate genes that could potentially be used as selective markers to finely control flowering in bean breeding programs.

Gali, Sackville et al. reported on studies using genome-wide association studies (GWAS) in field pea. They analyzed 135 pea accessions from a range of countries across all continents. The focus was on agronomic and seed related traits and the accessions were first characterized using genotyping-by-sequencing (GBS) from which a final set of 16,877 high quality SNPs were selected for marker-trait association analysis. This led to the identification of many SNPs with significant association with specific traits. In some cases, these mapped to QTLs previously associated with the specific trait. Overall, this large study generated resources that have potential use in marker-assisted selection for accelerating pea cultivar improvements.

Sanderson et al. described their online legume genetic and genomic resource, KnowPulse, which they have developed to serve legume breeding and genetic research communities. The database hosts phenotypic, genotypic and genomic data for chickpea, common bean, field pea, faba bean and lentil, which can be queried by a range of visualization and data exploration tools. Built on an open-source platform, it is amenable to community collaboration, which will help to ensure its ongoing relevance and usefulness. This kind of resource is vital for linking independent studies and deriving maximum value from costly datasets generated globally.

Legumes play an important role in the sustainability of agricultural and food systems, contributing to soil fertility and environmental protection, as well as to food safety and nutrition. Under this framework, the perception of *Lens culinaris* producers and consumers of North America has been evaluated by Warne et al., following agronomic, economic, and nutritional criteria. In a survey of producers, the main agroeconomic reason to introduce lentil in production systems was to diversify crop rotation in order to capitalize on dryland production and serve as a cash crop. Diversifying crop rotation improves agricultural system robustness, increasing system resistance to biotic stresses and resilience to abiotic disturbances favoring the constancy of crop productivity. According to consumers' perception, the main reasons to include lentil in their eating habits are to improve nutrition, the satiety feeling after intake and to support a plant-based diet. In agreement with that, lentils like other legumes are considered good sources of proteins, starch, fiber, vitamins, and minerals. Scientific evidence has demonstrated that carbohydrates resistant to digestion are the major factors responsible for both low glycaemic index of legume foods and consumers' feeling of satiety. Finally, lentils might take part as a component of a plant-based diet being an inexpensive and rich source of high-quality proteins to assure a balanced and healthy diet. Interestingly, the growing interest of consumers and non-consumers to increase lentil consumption seems to be based on environmental, economic and nutritional reasons. Suitable policy actions might help to address emerging challenges and concepts and open future opportunities in order to promote cultivation and increase lentil consumption.

The review manuscript by Ojiewo et al., looked at advances in research for nutritional quality and health benefits of groundnut (*Arachis hypogaea* L.). Groundnut is an important global crop both from a food point of view and for valuable levels of oil. This substantial review focused on breeding and genetic engineering approaches to improve various traits in groundnut including aflatoxin resistance, allergen issues and increasing oleic acid levels. The review also discussed important social approaches that are needed in this area and current progress including the ongoing efforts to improve distribution of good quality seed to small stakeholder farmers in many parts of the world.

Consumers of pulse crops in many markets highly value the appearance of the grain, with seed coat color and patterning being key traits. Herniter et al. investigated the genetics of seed coat patterning in cowpea using quantitative trait locus (QTL) and candidate gene approaches. They identified three loci with candidate genes (basic helix–loop–helix (bHLH), WD-repeat and E3 ubiquitin ligase genes) and developed a model to show how they interact to give the observed seed coat patterning.

Furthermore, Dakora and Belane identifying cowpea genotypes that can enhance protein accumulation and micronutrient density in edible leaves and seed through breeding has the potential to overcome protein-calorie malnutrition and trace element deficiency in rural Africa.

Taken together, the 36 articles reported in this special issue represent a substantial contribution to the advancement in our understanding and breeding of climate-resilient legumes, and we hope will lead to improved global food security in the longer term.

## Author Contributions

JJ-L, KS, AC, MN, SO, and PS have written, reviewed and edited the original draft. All authors have approved the final manuscript.

## Conflict of Interest

The authors declare that the research was conducted in the absence of any commercial or financial relationships that could be construed as a potential conflict of interest. The reviewer EW declared a past co-authorship with one of the authors MN to the handling editor.

